# Hypoglycemic and Hypolipidemic Potential of a High Fiber Diet in Healthy versus Diabetic Rabbits

**DOI:** 10.1155/2013/960568

**Published:** 2013-05-14

**Authors:** Raquel Díez, Juan J. García, M. José Diez, Matilde Sierra, Ana M. Sahagún, Ángela P. Calle, Nélida Fernández

**Affiliations:** Department of Pharmacology, Institute of Biomedicine (IBIOMED), University of León, 24071 León, Spain

## Abstract

The aim of this study was to investigate potential hypoglycaemic and hypolipidemic effects of Plantago ovata husk included in the diet, in healthy and diabetic rabbits. We also examined the effects of this fiber in other biochemical parameters. Two groups of 18 rabbits were used. The first group was fed with standard chow and the second with chow supplemented with Plantago ovata husk (3.5 mg/kg/day). On day 14 diabetes mellitus was induced by the intravenous administration of alloxan (80 mg/kg). After an oral glucose load (3 g), glucose, insulin, and other biochemical parameters were determined on day 14 (healthy rabbits) and on day 28 (diabetic rabbits). In healthy rabbits, fiber did not modify glucose or insulin levels but decreased significantly total cholesterol, LDL-cholesterol, atherogenic index, and glycosylated hemoglobin. In diabetic rabbits, fiber was more beneficial in mild diabetics than in severe diabetics with significant decreases in glucose levels and increases in insulin concentrations. In these animals fiber caused an important reduction in cholesterol, indicating a beneficial effect of Plantago ovata husk in diabetic rabbits. Although further studies in patients are necessary, we think that Plantago ovata husk offers interesting perspectives to be administered to patients with diabetes mellitus.

## 1. Introduction

Natural products have been a source of medicinal treatments for thousand of years, and plants-based systems continue to play an essential role in the primary health care of approximately 80% of the world's underdeveloped and developing countries [1].

Since Burkitt et al. [2] suggested that the lack of fiber might be the common origin of various diseases, its consumption has focused on the prevention and treatment of gastrointestinal disorders [3–5], some types of cancer [3, 6], hyperlipidemia [3, 7], cardiovascular diseases [3, 8], obesity [3, 9], and diabetes [3, 10, 11].

Viscous forms of dietary fiber have been shown to improve blood glucose control [12, 13] by trapping ingested carbohydrates inside the viscous gel formed after digestion. 

Psyllium or ispaghula husk (the husk of the seeds of *Plantago ovata*) is a mixture of neutral and acid polysaccharides with a rest of galacturonic acid. The polysaccharides are built up of the monomers D-xylose and L-arabinose, and ispaghula husk contains 67% pentosans. Ispaghula husk is a gel-forming (water-soluble) fiber whose benefits in terms of treatment for constipation are well established since many years ago.

Several authors have shown that this fiber reduces postprandial glucose concentrations [10, 12, 14–24]; however, others did not find any modification in postprandial glucose and insulin concentrations, neither in healthy subjects nor in type 2 diabetic patients [25].

Diabetes mellitus will be a major health problem in the 21st century because its prevalence is increasing worldwide. The prevalence of diabetes mellitus was estimated to be 177 million cases in 2000 and is projected to increase to 366 million by 2030, largely owing to an aging population, increased urbanization, and more sedentary lifestyles [26].

The important goal of diabetes mellitus treatment is to keep blood glucose, lipid, and lipoprotein levels close to normal, resulting in a reduction of coronary artery disease, a delay in onset, and a major slowing in the progression of complications [27]. Blood glucose control is of established benefit and remains a central tenet of long-term management for patients with diabetes mellitus. The risk of developing long-term complications can be substantially reduced with the implementation of intensive glycemic control.

Although sulphonylureas, biguanides, insulin sensitizers (thiazolidinediones), and other current drugs are valuable in the treatment of type 2 diabetes mellitus, their use is restricted by cost, limited pharmacokinetic properties, secondary failure rates, and accompanying side effects [28].

The purpose of this study was to investigate potential hypoglycaemic and hypolipidemic effects of Plantago ovata husk included in the diet, in healthy and diabetic rabbits. We also examined the effects of this fiber in other biochemical parameters such as uric acid, atherogenic index, iron, and calcium.

## 2. Material and Methods

### 2.1. Animals and Experimental Procedures

Thirty-six healthy male New Zealand white rabbits weighting 2.650–3.240 kg were used. The animals were housed in individual metal cages, which allowed the isolation of faeces in a lower container to avoid coprophagia. The environmental conditions were as follows: temperature 19 ± 2°C, relative humidity 55 ± 10%, and a 12 h light-dark cycle. Rabbits were maintained under these conditions at least 1 week before the assay, with free access to water and standard laboratory chow. The diet (compound feed for rabbits J-59, Alse) was specially prepared by Piensos Alse (León, Spain) by mixing the following ingredients: carbohydrate, 61.2%; protein, 14.5%; oils and fats, 3.8%; crude fiber, 12.8%; ash, 6.5%; calcium, 0.5%; phosphorus, 0.5%; sodium, 0.2%; vitamin, and trace elements.

The animals were randomized into two groups (groups 1 and 2) of 18 rabbits each. All the animals of the first group received standard chow and the rabbits of the second group received standard chow supplemented with Plantago ovata husk (the husk obtained from the seeds of *Plantago ovata*). This fiber was added to the chow to provide dose of 3.5 mg/kg/day to the rabbits.

The study began feeding the animals with the chow during two weeks. On day 14, and after an overnight fast, animals received an oral 3 g glucose load. After the administration, blood samples were collected at different times to obtain the glucose and insulin curves in healthy animals. A blood sample at time zero was also collected for the determination of the following biochemical parameters: glucose, total cholesterol, LDL-cholesterol, HDL-cholesterol, triglycerides, atherogenic index, glycosylated hemoglobin, uric acid, iron, calcium, phosphorus, magnesium chloride, sodium, and potassium. Blood samples (1 mL) were obtained from the marginal ear vein, using an intravenous catheter, at 0, 30, 60, 120, and 180 minutes after glucose administration. Immediately after collection, plasma was separated by centrifugation and stored at −20°C until analyzed.

At the end of the sampling collection, diabetes mellitus was induced by the intravenous administration of alloxan (80 mg/kg) dissolved in 10 mL NaCl solution in the marginal ear vein. Immediately, 2 mL dextrose 5% was injected, and this administration was repeated at 20 minutes, 4, 6, and 8 hours after alloxan injection.

Blood glucose concentrations were monitored for two weeks until diabetes was stabilized. After diabetes stabilization and an overnight fast, on day 28, the rabbits received a new 3 g oral glucose load, and the determination of glucose and insulin concentrations repeated. As well, the same biochemical parameters were evaluated.

All studies were performed in accordance with the Spanish Regulations for the handling and use of laboratory animals (RD 1201/2005). Minimum number of animals and duration of observation required to obtain consistent data were employed.

The measurement of glucose and the other biochemical parameters was carried out by using a biochemical autoanalyzer (Cobas Integra 400). Insulin was determined by a radiometric method using a kit (Mercodia Ultrasensitive Insulin ELISA, Biosource Europe, SA).

Arithmetic means, SDs, and CVs were calculated from the results measured. Areas under the concentration (AUC) curves were calculated by trapezoidal rule for blood glucose and insulin concentrations from time zero to the last determined sample time. Maximum plasma glucose and insulin concentration (*C*_max⁡_) and the time to reach maximum concentration (*t*_max⁡_) were read directly from the individual plasma concentration-time curves.

*Statistical Analysis.* The data obtained for the two groups (animals receiving standard chow or supplemented with Plantago ovata husk) were compared for statistical significance by *t*-test, at *P* ≤ 0.05. All analyses were performed by using SPSS Statistics 17.0 for Windows.

## 3. Results

### 3.1. Serum Glucose

**Figure 1:**
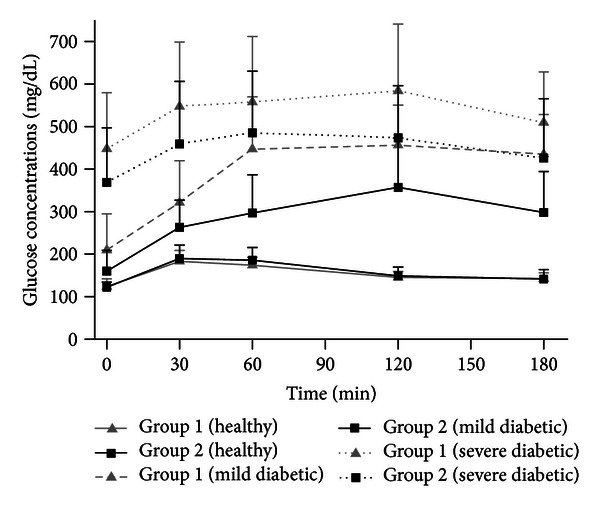
Mean ± SD plasma glucose concentrations in healthy, mild diabetic, and severe diabetic rabbits after the administration of an oral 3 g glucose load with standard chow (group 1) or supplemented chow (group 2).

The values of the mean plasma glucose concentrations as a function of time obtained after the oral administration of a 3 g glucose load on day 14 (healthy rabbits) to animals fed with standard chow (group 1) or supplemented with Plantago ovata husk (group 2) are shown in Figure 1. In this figure, glucose mean plasma concentrations obtained on day 28 (mild and severe diabetic animals) can be also seen. On day 28, when diabetes was stabilized, the animals were divided in two groups: severe diabetics and mild diabetics. The rabbits showing fasting blood glucose values 250 mg/dL or above were considered as severe diabetic rabbits group and those with values between 120 and 250 mg/dL were considered as mild diabetic rabbits group [29].

In healthy rabbits (day 14), the presence of fiber in the chow did not modify glucose concentrations, being very similar in both groups. When the rabbits were made diabetics (day 28) fiber caused a more important reduction in concentrations, higher in mild diabetic animals as shown in the figure. There were no significant differences between groups 1 and 2 (day 14) in these parameters for healthy animals.

Table 1 includes the values of *t*_max⁡_, *C*_max⁡_, and AUC determined for glucose in group 1 (standard chow) and group 2 (fiber supplemented) on day 14 (healthy rabbits) as well as on day 28 (mild and severe diabetic rabbits). 

The presence of fiber in the chow (day 28) reduced the values of *C*_max⁡_ and AUC in both mild and severe diabetic rabbits. This reduction was more important in mild diabetic rabbits, being 21.7% for *C*_max⁡_ and 26.3% for AUC (significant differences, *t*-test, *P* ≤ 0.05) and lower in severe diabetic animals (9.6% for *C*_max⁡_ and 11.2% for AUC) with no significant differences. It was also observed that *t*_max⁡_ was reached before (13.3 min. in mild diabetics and 21 min. in severe diabetics) although differences were not significant.

### 3.2. Serum Insulin

**Figure 2:**
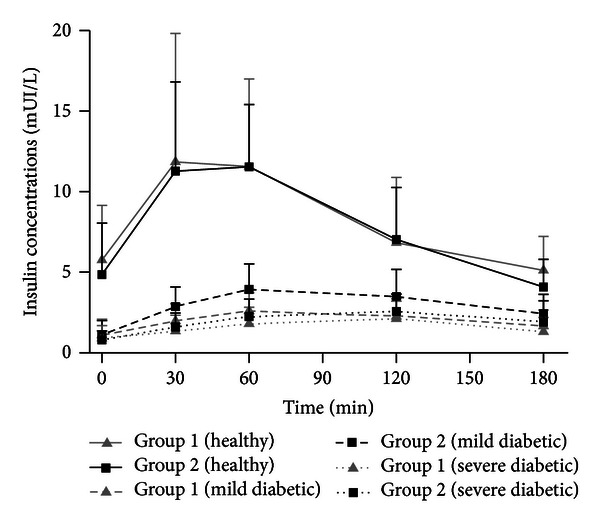
Mean ± SD plasma insulin concentrations in healthy, mild diabetic, and severe diabetic rabbits after the administration of an oral 3 g glucose load with standard chow (group 1) or supplemented chow (group 2).

Figure 2 shows the values of the mean plasma insulin concentrations as a function of time obtained after the oral administration of a 3 g glucose load to rabbits fed with standard chow (group 1) or supplemented with Plantago ovata husk (group 2) on day 14 (healthy animals) and on day 28 (diabetic rabbits). The presence of fiber in chow did not modify insulin levels in healthy rabbits; however, in diabetic animals fiber caused an increase in insulin concentrations, higher in mild diabetics.

Table 2 includes the values of *t*_max⁡_, *C*_max⁡_, and AUC determined for insulin in group 1 and group 2 on day 14. As can be seen in this table, in healthy rabbits, the inclusion of Plantago ovata husk in the chow reduced by 26.5% the value of *C*_max⁡_, although differences were not significant. AUC was also lower in the presence of fiber (19.7%), and *t*_max⁡_ was reached later (8 min.), although these differences were not significant.

The changes caused by the presence of fiber in the feeding of mild diabetic rabbits were more important than in severe diabetic rabbits. There was a significant increase (*t*-test, *P* ≤ 0.05) in *C*_max⁡_ (50.7%) and in AUC (51.7%) with no modification in *t*_max⁡_ for mild diabetic rabbits. In severe diabetics, these increases were much lower (5.7% and 7.4%, resp., for *C*_max⁡_ and AUC). The value of *t*_max⁡_ was not modified by the presence of Plantago ovata husk and was higher than that in mild diabetics.

### 3.3. Other Biochemical Parameters

The values of different biochemical parameters evaluated in healthy rabbits (day 14) are included in Table 3.

All parameters were lower in group 2 (fiber included in chow) than in group 1 (standard diet), although the differences only were significant for total cholesterol, LDL-cholesterol, atherogenic index, and glycosylated hemoglobin (*t*-test, *P*≤ 0.05). 

Biochemical parameters values for mild diabetic rabbits are shown in Table 4. In these animals, all values, except iron, were lower in animals fed with chow supplemented with fiber, being the differences significant for total cholesterol, LDL-cholesterol, HDL-cholesterol, triglycerides, glycosylated hemoglobin, and uric acid (*t*-test, *P*≤ 0.05). Triglycerides (48.7%) and uric acid (45.9%) were the parameters that showed the highest reduction.

Table 5 includes the values for these biochemical parameters in severe diabetic rabbits. In this group of animals, the decreases observed in all parameters in supplemented chow group were higher, being the highest decreases for uric acid (66%) and total cholesterol (42.6%). There were significant differences in total cholesterol, LDL-cholesterol, HDL-cholesterol, triglycerides, atherogenic index, glycosylated hemoglobin, uric acid, phosphorus, and magnesium (*t*-test, *P*≤ 0.05).

## 4. Discussion

### 4.1. Glucose

According to Quesenberry and Carpenter [30] and Kahn [31], rabbit's fasting glucose ranges from 75 mg/dL to 155 mg/dL, values very similar to those found in this study, varied from 79.4 mg/dL to 156.0 mg/dL. The presence of fiber (Plantago ovata husk) in the chow of these healthy rabbits modified significantly neither basal nor postprandial glucose levels although a slight reduction was observed.

In the bibliographical review, we have not found any study regarding the effect of dietary fiber on glucose levels in healthy rabbits. However, the results obtained in other experimental animals were similar to ours. Thus, in sows, after the inclusion of a fermentable dietary fiber, postprandial glucose levels were reduced without modifying its basal concentrations [32]. Adding purified soluble (pectin) or insoluble (lignocellulose) fiber to a corn meal did not affect postprandial glucose responses in healthy horses, while AUC and *C*_max⁡_ values were slightly higher with this meal [33]. In healthy volunteers, the results are variable. In this way, Sierra et al. [23] showed that the administration of Plantago ovata husk may be beneficial due to its ability to reduce glucose postprandial concentrations. The authors find that the AUC curve obtained for glucose after the administration of a 50 g glucose load was reduced by 11.1% (significant differences) in the presence of Plantago ovata husk and 2.6% with gum guar (no significant differences). Aller et al. [34] suggested that a modest increase in soluble fiber intake in healthy subjects for 3 months improved glucose levels. Other study [35] demonstrated that the addition of a highly viscous fiber to a starchy snack during 2 weeks is able to reduce the glycemic response in healthy volunteers [36], and Kim et al. [37] also suggested that glucose response was not significantly different when adding *β*-glucan to meal.

Several studies have been carried out using experimental alloxan-induced diabetic rabbits. In these animals, fasting glucose was determined, being the values obtained very similar to ours. Thus, Annamala and Augusti [38] showed average values of 329.1 mg/dL and Lenich et al. [39] determined glucose values between 368 and 380 mg/dL. Schiller and McNamara [40] considered as hyperglycaemic rabbits those with basal glucose between 170 and 400 mg/dL and diabetics when values were above 400 mg/dL. Fleitas et al. [41] obtained basal values for glucose above 252 mg/dL 2 days after alloxan-induced diabetes and Godwin et al. [42] indicated a mean value of 334.0 mg/dL.

In the present study, the results obtained showed that the inclusion of Plantago ovata husk in the feeding of diabetic rabbits was more beneficial in mild diabetics than in severe diabetics with significant decreases in glucose levels.

Other authors showed that the inclusion of this soluble fiber in food of diabetic mice significantly reduced glucose basal levels in relation to mice that were fed without fiber [43]. In type 2 diabetic patients, supplemented with 5.1 g of psyllium or cellulose placebo twice daily for 8 weeks, other authors found postlunch postprandial glucose concentration was 19.2% lower in the psyllium than in the placebo group [21]. Sierra et al. [11] evaluated the effects of psyllium in type 2 diabetic patients. All volunteers received 3.5 g of psyllium four times a day during 6 weeks and the last day, after overnight fast, ingested a standard breakfast. Before breakfast, one dose of the fiber was given. It was demonstrated that glucose absorption decreased significantly in the presence of psyllium (12.2%). Ziai et al. [44] showed that 8 weeks of treatment with 5.1 g of psyllium twice a day can reduce plasma glucose and fluctuations in blood glucose control.

It was observed that other fibers also modified glucose response. In this way, another author after glucose load in diabetic rats with soybean fiber chow found significantly delayed *t*_max⁡_ against control group [45]. Similarly, 10–20% guar gum supplemented diets significantly decreased glucose plasma concentrations in diabetic rats [46].

Guar gum and wheat bran dietary inclusion for 2 months reduced significantly urinary glucose excretion and fasting glucose in type 2 diabetic patients [47].

### 4.2. Insulin

Schiller and McNamara [40] found basal mean insulin concentrations of 33 mUI/L, higher than those found in our rabbits. The basal values determined by Kawai et al. [48] were 3.82 mUI/L. Our results showed that the presence of fiber in the chow of healthy rabbits did not modify insulin levels. As occurred with glucose, we did not found any work about dietary fiber's effects in insulin levels from healthy rabbits. 

In sows, De Leeuw et al. [32] concluded that sugarbeet pulp, as a source of fermentable dietary fiber twice a day, did not change basal insulin levels but stabilized postprandial levels and reduced physical activity in limited-fed sows several hours after feeding. Adding purified soluble (0.1 g/kg/day) or insoluble fiber (0.2 g/kg/day) to a corn meal during 10 days did not affect postprandial insulin response in healthy horses [33]. According to the results obtained for Sierra et al. [23] in healthy women, the administration of glucose (50 g) and Plantago ovata husk (10.5 g/day) after an overnight fast during 2 days may be beneficial to reduce insulin requirements. In this study, insulin AUC was reduced by 36.1% when ispaghula husk was administered, and accumulated AUC values were lower when fiber was administered from 30 up to 120 min. Wolever et al. [49] observed in men that plasma insulin was significantly lower after the administration of a high-fiber cereal meal than after giving a low-fiber cereal.

Flood et al. [50] analyzed fasting insulin serum from 750 subjects with (375) and without (375) a low-fat, high-fiber, high-fruit, and -vegetable dietary after 4 years, showing that this intervention had minimal impact on serum concentrations of insulin. Kim et al. [37] suggest that acute consumption of 10 g of *β*-glucan is able to induce physiologically beneficial effects on postprandial insulin responses in obese women at risk for insulin resistance.

In alloxan-induced diabetic rabbits, Schiller and McNamara [40] obtained mean baseline insulin values of 9.3 mUI/L. This value is much higher than those obtained in this study (0.464–1.412 mUI/L). Other authors [51] obtained mean baseline insulin values of 6.24 mUI/L.

The inclusion of Plantago ovata husk in the feeding of diabetic rabbits was more beneficial in mild diabetic (significant differences) than in severe diabetic rabbits.

Insulin levels were significantly higher in psyllium-fed than in placebo-fed mice, indicating that this fiber may delay the progression of diabetes in the animal model [43]. Madar [45] studied the effect of brown rice (10%) and soybean (10%) dietary fiber on the oral glucose tolerance test in diabetic rats and observed that insulin levels were not affected by the inclusion of fiber in diet. The same conclusion was achieved by Ray et al. [47] after giving a diet with 20 g of guar gum during 2 months to 12 obese and poor controlled noninsulin-dependent diabetic patients. However, Sierra et al. [11] observed changes in insulin levels (reduction 5%) in 20 type 2 diabetic patients that receieved 3.5 g of psyllium four times a day.

The present study shows that the presence of the fiber in the chow modified glucose and insulin levels in diabetic rabbits. The possible mechanism of action of this gel-forming fiber is related to the ability to increase the viscosity of the gastrointestinal contents, and thus, interfering with motility and absorption [52].

### 4.3. Other Biochemical Parameters

Our parameters showed that the values obtained in group 2 were lower than those found in group 1. In a study carried out to develop experimental diabetes model in healthy rabbits [53] the authors found average values for total cholesterol and HDL-cholesterol slightly lower than those reported in our study, while triglycerides were higher. The values of total cholesterol and HDL-cholesterol reported by Sharma et al. [29] were similar to ours, while LDL-cholesterol was lower.

In diabetic animals, minerals, sodium, and potassium were within the range indicated by Quesenberry and Carpenter [30], while calcium and phosphorus values were slightly higher.

In severe diabetic rabbits, total cholesterol values were similar to those found by other authors [38], while those of LDL-cholesterol were significantly lower and HDL-cholesterol levels much higher.

## 5. Conclusions

In conclusion, the inclusion of Plantago ovata husk in the chow caused an important reduction in glucose levels and an important increase in insulin levels in mild diabetic rabbits. A hypolipidemic effect was also observed.

Although further studies in patients are necessary, we think that Plantago ovata husk offers interesting perspectives to be administered to patients with diabetes mellitus. The administration of this fiber can reduce glucose and increase insulin levels and also improve other parameters, mainly cholesterol that usually shows high levels in diabetics.

## Figures and Tables

**Table 1:** Pharmacokinetic parameters for glucose obtained on days 14 (healthy rabbits) and 28 (diabetic rabbits) after the administration of an oral 3 g glucose load.

	Day 14 (healthy rabbits)				
	Standard chow		Supplemented chow		Standard/supplemented variation
	$\overset{-}{X}$ ± SE	CV (%)	$\overset{-}{X}$ ± SE	CV (%)	
*t*_max⁡_ (min)	30.00 ± 3.55	36.12	30.00 ± 3.02	35.00	
*C*_max⁡_ (mg/dL)	186.1 ± 5.43	12.37	189.7 ± 9.66	20.60	↑ 3.6%
AUC (mg · min/dL)	28168.2 ± 689.1	10.63	29539.0 ± 1122.1	16.82	↑ 4.9%

	Day 28 (mild diabetic rabbits)				
	Standard chow		Supplemented chow		Standard/supplemented variation
	$\overset{-}{X}$ ± SE	CV (%)	$\overset{-}{X}$ ± SE	CV (%)	

*t*_max⁡_ (min)	95.00 ± 11.65	34.68	81.67 ± 10.19	39.46	
*C*_max⁡_ (mg/dL)	456.2 ± 26.31^1^	15.80	357.1 ± 23.70	27.38	↓ 21.7%
AUC (mg · min/dL)	73304.3 ± 4939.9^1^	18.86	54008.6 ± 3889.8	16.82	↓ 26.3%

	Day 28 (severe diabetic rabbits)				
	Standard chow		Supplemented chow		Standard/supplemented variation
	$\overset{-}{X}$ ± SE	CV (%)	$\overset{-}{X}$ ± SE	CV (%)	

*t*_max⁡_ (min)	118.2 ± 13.24	34.68	97.25 ± 12.87	39.46	
*C*_max⁡_ (mg/dL)	583.8 ± 32.43	15.80	527.7 ± 31.70	27.38	↓ 9.6%
AUC (mg · min/dL)	98530.0 ± 6556.6	18.86	87486.5 ± 6229.9	27.81	↓ 11.2%

^1^Significative differences (*t*-test, *P* ≤ 0.05).

**Table 2:** Pharmacokinetic parameters for insulin obtained on days 14 (healthy rabbits) and 28 (diabetic rabbits) after the administration of an oral 3 g glucose load.

	Day 14 (healthy rabbits)				
	Standard chow		Supplemented chow		Standard/supplemented variation
	$\overset{-}{X}$ ± SE	CV (%)	$\overset{-}{X}$ ± SE	CV (%)	
*t*_max⁡_ (min)	33.33 ± 2.14	29.10	41.67 ± 3.55	36.12	
*C*_max⁡_ (mUI/dL)	18.30 ± 1.82	42.22	13.45 ± 1.39	43.79	↓ 26.5%
AUC (mUI · min/dL)	1779.7 ± 164.9	39.33	1428.3 ± 141.2	42.08	↓ 19.7%

	Day 28 (mild diabetic rabbits)				
	Standard chow		Supplemented chow		Standard/supplemented variation
	$\overset{-}{X}$ ± SE	CV (%)	$\overset{-}{X}$ ± SE	CV (%)	

*t*_max⁡_ (min)	60.00 ± —	—	60.00 ± —	—	
*C*_max⁡_ (mUI/dL)	2.59 ± 0.51^1^	68.08	3.93 ± 0.44	61.82	↑ 50.7%
AUC (mUI · min/dL)	117.5 ± 20.37^1^	66.22	177.1 ± 26.29	58.91	↑ 51.7%

	Day 28 (severe diabetic rabbits)				
	Standard chow		Supplemented chow		Standard/supplemented variation
	$\overset{-}{X}$ ± SE	CV (%)	$\overset{-}{X}$ ± SE	CV (%)	

*t*_max⁡_ (min)	120.00 ± —	—	120.00 ± —	—	
*C*_max⁡_ (mUI/dL)	2.11 ± 0.40	68.08	2.23 ± 0.38	61.82	↑ 5.7%
AUC (mUI · min/dL)	102.1 ± 19.05	66.22	109.7 ± 20.21	58.91	↑ 7.4%

^1^Significative differences (*t*-test, *P* ≤ 0.05).

**Table 3:** Biochemical parameters obtained on day 14 (healthy rabbits).

Healthy rabbits	Standard chow ($\overset{-}{X}$ ± SE)	Supplemented chow ($\overset{-}{X}$ ± SE)	Standard/supplemented variation
Cholesterol (mg/dL)	68.31 ± 5.33	58.40 ± 6.14	↓ 14.5%^1^
LDL-cholesterol (mg/dL)	46.45 ± 3.14	39.71 ± 3.61	↓ 14.5%^1^
HDL-cholesterol (mg/dL)	33.24 ± 1.44	32.06 ± 1.63	↓ 3.5%
Triglycerides (mg/dL)	85.50 ± 8.39	77.65 ± 6.49	↓ 9.2%
Atherogenic index	2.06 ± 0.16	1.66 ± 0.12	↓ 19.1%^1^
Uric acid (mg/dL)	0.11 ± 0.02	0.11 ± 0.01	—
Glycosylated hemoglobin (%)	2.93 ± 0.40	2.31 ± 0.27	↓ 21.0%^1^
Ca (mg/dL)	13.36 ± 0.16	13.24 ± 0.16	↓ 0.9%
P (mg/dL)	8.75 ± 0.13	8.16 ± 0.06	↓ 6.7%
Mg (mg/dL)	2.76 ± 0.10	2.67 ± 0.10	↓ 3.3%
Cl (mg/dL)	100.43 ± 0.87	—	—
Na (mg/dL)	142.52 ± 0.85	142.95 ± 0.90	↓ 0.3%
K (mg/dL)	4.32 ± 0.10	4.09 ± 0.08	↓ 5.3%
Fe (mg/dL)	120.0 ± 2.83	122.95 ± 8.23	↓ 2.5%

^1^Significative differences (*t*-test, *P* ≤ 0.05).

**Table 4:** Biochemical parameters obtained on day 28 (mild diabetic rabbits).

Mild diabetic rabbits	Standard chow ($\overset{-}{X}$ ± SE)	Supplemented chow ($\overset{-}{X}$ ± SE)	Standard/supplemented variation
Cholesterol (mg/dL)	84.83 ± 18.04	70.25 ± 11.84	↓ 17.2%^1^
LDL-cholesterol (mg/dL)	57.70 ± 3.92	47.80 ± 2.81	↓ 20.6%^1^
HDL-cholesterol (mg/dL)	22.67 ± 2.93	16.0 ± 2.64	↓ 20.6%^1^
Triglycerides (mg/dL)	289.0 ± 20.45	148.25 ± 19.41	↓ 48.7%^1^
Atherogenic index	3.24 ± 0.36	3.07 ± 0.62	↓ 5.2%
Uric acid (mg/dL)	0.16 ± 0.02	0.09 ± 0.02	↓ 45.9%^1^
Glycosylated hemoglobin (%)	6.41 ± 0.04	4.80 ± 0.02	↓ 25.1%^1^
Ca (mg/dL)	12.54 ± 0.32	12.04 ± 0.32	↓ 4.0%
P (mg/dL)	7.78 ± 0.13	6.26 ± 0.11	↓ 19.5%
Mg (mg/dL)	2.38 ± 0.09	2.14 ± 0.12	↓ 10.1%
Cl (mg/dL)	88.0 ± 6.24	—	—
Na (mg/dL)	136.0 ± 0.85	136.20 ± 1.06	↑ 0.1%
K (mg/dL)	3.86 ± 0.42	3.53 ± 0.06	↓ 8.6%
Fe (mg/dL)	67.0 ± 4.14	80.67 ± 7.43	↑ 17.0%

^1^Significative differences (*t*-test, *P* ≤ 0.05).

**Table 5:** Biochemical parameters obtained on day 28 (severe diabetic rabbits).

Severe diabetic rabbits	Standard chow ($\overset{-}{X}$ ± SE)	Supplemented chow ($\overset{-}{X}$ ± SE)	Standard/supplemented variation
Cholesterol (mg/dL)	317.18 ± 32.97	182.09 ± 15.60	↓ 42.6%^1^
LDL-cholesterol (mg/dL)	215.70 ± 17.23	123.80 ± 9.70	↓ 31.6%^1^
HDL-cholesterol (mg/dL)	29.79 ± 3.46	20.38 ± 3.09	↓ 31.6%^1^
Triglycerides (mg/dL)	715.97 ± 128.56	527.43 ± 89.87	↓ 26.3%^1^
Atherogenic index	8.71 ± 1.67	6.90 ± 0.45	↓ 20.8%^1^
Uric acid (mg/dL)	0.23 ± 0.03	0.08 ± 0.02	↓ 66.0%^1^
Glycosylated hemoglobin (%)	6.46 ± 0.05	4.93 ± 0.05	↓ 23.6%^1^
Ca (mg/dL)	12.86 ± 0.54	11.70 ± 0.52	↓ 9.0%
P (mg/dL)	8.22 ± 0.27	6.0 ± 0.19	↓ 27.0% ^1^
Mg (mg/dL)	2.84 ± 0.09	2.15 ± 0.08	↓ 24.3% ^1^
Cl (mg/dL)	83.40 ± 5.63	—	—
Na (mg/dL)	133.22 ± 8.31	137.22 ± 5.64	↓ 3.0%
K (mg/dL)	3.54 ± 0.05	3.44 ± 0.04	↓ 2.8%
Fe (mg/dL)	87.10 ± 4.30	102.0 ± 4.13	↓ 14.6%

^1^Significative differences (*t*-test, *P* ≤ 0.05).
